# Radial tunnel syndrome in psoriatic enthesitis: Complication or coincidence?

**DOI:** 10.31138/mjr.32.4.373

**Published:** 2021-12-27

**Authors:** Nayan Patel Sureja

**Affiliations:** Department of Rheumatology and Clinical Immunology, Star Hospitals, Hyderabad, India

**Keywords:** Enthesitis, psoriasis, psoriatic arthritis, radial neuropathy

## Abstract

Radial tunnel syndrome (RTS) is a rare condition resulting from posterior interosseous nerve (a branch of radial nerve) compression, within the radial tunnel. Lateral epicondylitis as a possible aetiology for RTS has been previously described. Here we report a 64-year-old female, with history of scalp psoriasis, who presented with pain over the lateral aspect of the left elbow and proximal forearm for one year, and decreased sensation over the lateral aspect of distal left forearm including the hand for 20 days. Examination revealed enthesitis at left lateral epicondyle, which was confirmed on magnetic resonance imaging. Touch and pain sensations were reduced over the left thumb, index finger, lateral aspect of the hand and distal forearm, and forearm pain was exaggerated with restricted extension of the wrist and third finger. Rheumatoid factor, anti-nuclear and anti-neutrophil cytoplasmic antibody were negative, and nerve conduction study were normal. A diagnosis of RTS as a possible complication of psoriatic enthesitis was suggested, and patient showed good response to non-steroidal anti-inflammatory drugs.

## INTRODUCTION

Radial tunnel syndrome (RTS) is usually described as a rare condition resulting from posterior interosseous nerve (a branch of radial nerve) compression, within the radial tunnel. Descriptions about RTS in the literature are ambiguous. Possible etiological factors for RTS are trauma, tumours with mass effect, and elbow arthritis.^1^ Lateral epicondylitis as a possible aetiology for RTS has been described only once.^2^ Here, we report a case of RTS in a patient with psoriatic enthesitis.

## CASE REPORT

**Figure 1.**
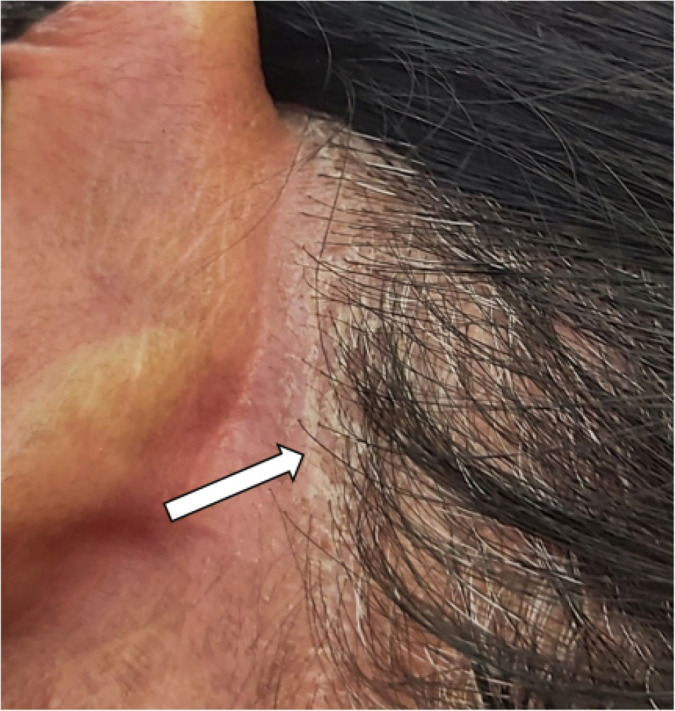
Psoriatic skin lesion behind the left ear (arrow).

A 64-year-old female with history of scalp psoriasis for more than 30 years, presented with pain over the lateral aspect of the left elbow and proximal aspect of the left forearm for one year, and decreased sensation over the lateral aspect of distal left forearm including the hand for 20 days. Physical examination showed psoriatic lesions over the scalp (**Figure 1,** arrow). Tenderness was elicited over the left lateral epicondyle and five centimetres distal to it. Forearm pain was exaggerated with restricted extension of the wrist and third finger. Touch and pain sensations were reduced over the left thumb, index finger, lateral aspect of the hand and distal forearm. Power at all the joints including interossei, lumbericals, thenar, and hypothenar muscles was normal.

**Figure 2.**
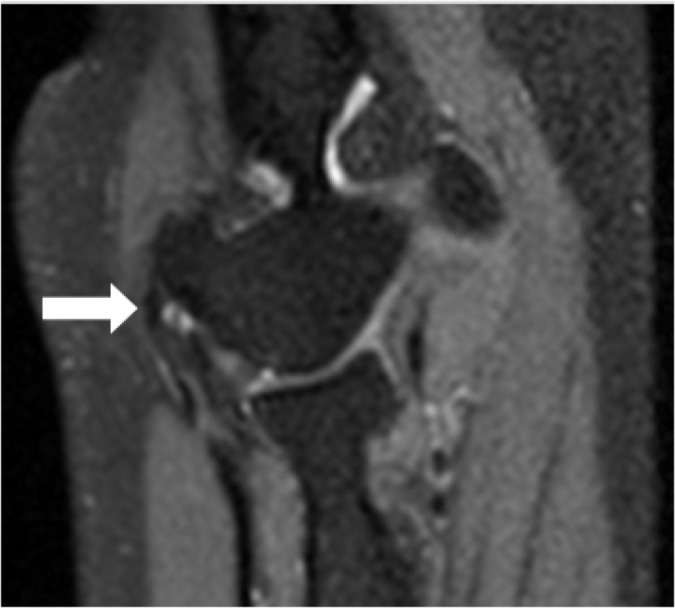
3D proton density fat saturated coronal magnetic resonance image of the left elbow showing fluid signal at the insertion of common extensor tendon on the lateral epicondyle, suggestive of enthesitis (arrow).

On evaluation, erythrocyte sedimentation rate and C-reactive protein were normal. Rheumatoid factor, anti-nuclear and anti-neutrophil cytoplasmic antibody were negative. Radiographs of the left elbow, electromyography (EMG), and nerve conduction velocity (NCV) studies were normal. Magnetic resonance imaging (MRI) confirmed enthesitis at left lateral epicondyle (**Figure 2,** arrow). A diagnosis of RTS as a possible complication of psoriatic enthesitis was made, and patient showed good response to non-steroidal anti-inflammatory drugs.

## DISCUSSION

Radial tunnel is a space in the proximal forearm, extending from the radiocapitellar joint to the distal margin of supinator muscle, bordered laterally by the brachioradialis, extensor carpi radialis brevis (ECRB) and longus muscles, and medially by the biceps and brachialis muscles.^2^ The possible sites of radial nerve compression in this tunnel are; the capsular tissue of the radiocapitellar joint, leash of Henry (an arcade of branches of the recurrent radial artery), the fibrous edge of ECRB, the Frohse arcade (formed by the proximal border of superficial head of supinator muscle), and the distal edge of supinator muscle.^1^

RTS presents with pain in the dorsolateral aspect of the proximal forearm. Sometimes sensory disturbances are also found along lateral aspect of the hand and distal forearm, without any motor weakness. RTS is diagnosed solely on the clinical signs after excluding other aetiologies. The closest differential diagnosis is lateral epicondylitis, where the site of tenderness is on the lateral epicondyle. Whereas, tenderness in RTS is elicited around five centimetres distal to the lateral epicondyle.^1^ However, both these conditions can coexist,^3^ and lateral epicondylitis has also been considered as an etiological factor for RTS.^2^

Enthesitis is inflammation of tendons and ligaments at their insertion into bone. It is the hallmark of psoriatic arthritis (PsA), with a prevalence of 35–50%.^4^ Enthesitis is one of the entry components of the CASPAR (ClASsification for Psoriatic Arthritis) criteria.^5^ Lateral epicondylitis/enthesitis in the present case was an early manifestation of PsA disease spectrum, and RTS was possibly a complication of this process. However, the possibility of these two conditions coexisting cannot be excluded. Further observations are required to strengthen the association of RTS and lateral epicondylitis/psoriatic enthesitis.

Making a diagnosis of RTS remains a difficult task due to lack of confirmatory tests. Although MRI, EMG and NCV are usually normal in these patients, these helps in excluding other aetiologies of the elbow/proximal forearm pain. Initial step of management of RTS remains non-surgical methods which include anti-inflammatory medications, physical therapy, wrist immobilisation using a splint or cast, and modification of activities such as avoiding wrist flexion, forearm pronation and prolonged elbow extension. Surgical decompression is the next option if there is no response to these non-surgical treatment methods.^6^

The present case emphasises on the importance of determining the exact location of pain by detailed clinical examination in a patient with elbow and/or proximal forearm pain.

## CONSENT

Written informed consent was obtained from the patient.
